# PeakSeeker: a program for interpreting genotypes of mononucleotide repeats

**DOI:** 10.1186/1756-0500-2-17

**Published:** 2009-02-03

**Authors:** James M Thompson, Stephen J Salipante

**Affiliations:** 1 Department of Genome Sciences, University of Washington School of Medicine, Seattle, USA

## Abstract

**Background:**

Mononucleotide repeat microsatellites are abundant, highly polymorphic DNA sequences, having the potential to serve as valuable genetic markers. Use of mononucleotide microsatellites has been limited by their tendency to produce "stutter", confounding signals from insertions and deletions within the mononucleotide tract that occur during PCR, which complicates interpretation of genotypes by masking the true position of alleles. Consequently, microsatellites with larger repeating subunits (dinucleotide and trinucleotide motifs) are used, which produce less stutter but are less genetically heterogeneous and less informative. A method to interpret the genotypes of mononucleotide repeats would permit the widespread use of those highly informative microsatellites in genetic research.

**Findings:**

We have developed an approach to interpret genotypes of mononucleotide repeats using a software program, named PeakSeeker. PeakSeeker interprets experimental electropherograms as the most likely product of signals from individual alleles. Because mononucleotide tracts demonstrate locus-specific patterns of stutter peaks, this approach requires that the genotype pattern from a single allele is defined for each marker, which can be approximated by genotyping single DNA molecules or homozygotes. We have evaluated the program's ability to discriminate various types of homozygous and heterozygous mononucleotide loci using simulated and experimental data.

**Conclusion:**

Mononucleotide tracts offer significant advantages over di- and tri-nucleotide microsatellite markers traditionally employed in genetic research. The PeakSeeker algorithm provides a high-throughput means to type mononucleotide tracts using conventional and widely implemented fragment length polymorphism genotyping. Furthermore, the PeakSeeker algorithm could potentially be adapted to improve, and perhaps to standardize, the analysis of conventional microsatellite genotypes.

## Background

Microsatellites are short (1- to 5-bp), tandemly repeated DNA motifs that are useful as genetic markers because they display a high degree of polymorphism within populations [1-3]. Polymorphisms consist of differences in the number of repeat sequences contained by a microsatellite and are the consequence of mutations which occur during DNA replication, when subunits are inserted or deleted [4]. Although the mutation rate of microsatellites are influenced by a variety of factors [5,6], they tend to be inversely proportional to the length of the repeat unit [7,8]. Accordingly, mononucleotide repeats, uninterrupted tracts of A/T or G/C, are most susceptible to mutation [8] and are the most polymorphic [1] class of microsatellite. Polymorphisms at those sites are detectable even among somatic cells from the same individual [9,10].

**Figure 1:**
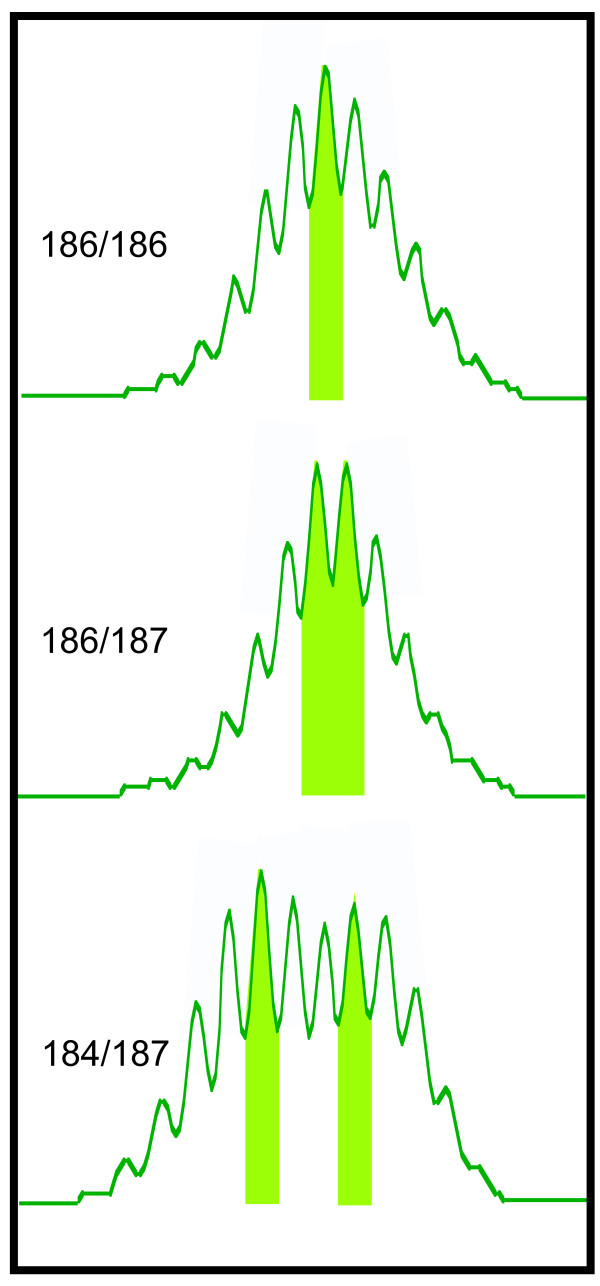
**Examples of genotypes from a polymorphic mononucleotide repeat in mouse**. PCR fragment size is represented on the X-axis (bp), intensity of fluorescent signal on the Y-axis (arbitrary units). Allele sizes are reported in bp, and their corresponding positions highlighted in the electropherograms. All non-indicated peaks represent "stutter" artifact. Electropherograms from [9].

Despite this desirable property, di-, tri-, and tetranucleotide repeat microsatellites are conventionally used. This discrepancy results from the technology used to evaluate microsatellite polymorphisms, fragment length polymorphism genotyping, in which microsatellite repeats are PCR amplified using fluorescently labeled primers and resolved by capillary electrophoresis. The examination of mononucleotide microsatellites by that approach is complicated by the production of excessive "stutter" (Figure 1), PCR products of varying sizes that arise from insertions or deletions within the mononucleotide tract during PCR amplification [11]. These additional signals complicate interpretation of electropherograms by masking the true positions of alleles [12], especially for diploid organisms, where stutter from heterozygous alleles may overlap and produce complex signals. As a result, most studies rely on microsatellites composed of longer repeating subunits, which produce less stutter during genotyping and are simpler to interpret, but which are also less genetically heterogeneous and require the examination of more markers to obtain the same amount of information. Analysis of mononucleotide microsatellites has been limited to the qualitative observation of instability [13,14], or quantitation of relatively large, and easier to detect changes [15]. However, because most mutations at mononucleotide tracts occur as insertions or deletions of only one base [1,8], such approaches are insensitive to common polymorphisms, and their application has been limited to examining microsatellite instability in tumors [6,14,16-20], where large mutations are unusually abundant.

Stutter artifacts also complicate determining tract lengths by DNA sequencing [11], and even next-generation genomic sequencing technologies experience problems at mononucleotide runs [21]. Although dedicated methods have been developed to detect single-base length differences in mononucleotide repeats, including mass-spectrometry [22] and primer-extension PCR [1,23], none has come into common use.

The accessibility of highly informative mononucleotide microsatellites could be improved by a high-throughput means to detect single-base length polymorphisms using fragment length polymorphism genotyping, which is already in widespread use. Here we describe an approach to interpret the genotypes of mononucleotide repeats with the aid of an analysis algorithm, which we have named PeakSeeker.

### Algorithm

PeakSeeker [Additional File 1: **PeakSeeker_V1.zip**] operates by interpreting the genotype of an autosomal mononucleotide repeat as the additive product of two homozygous genotypes, each corresponding to one of the two alleles. The program considers each homozygous and heterozygous combination of the two alleles' genotypes over the range where experimental data are present, and the relative amplification at each position is varied such that the additive product of the two allelic genotypes best fits the interrogated data. PeakSeeker scores each potential interpretation of the experimental genotype given how well the additive product fits the experimental data, and how realistic the required degree of unequal allelic amplification. The interpretation with the best score is reported, corresponding to the most probable interpretation. In order to reduce "noise" from stochastic variability between genotypes [9], the program can average together data from replicate genotypes and produce a "consensus" used for the analysis.

For full description of the program's workflow and scoring mechanism see [Additional File 2: **MethodsSupplement.pdf**].

### Testing

#### (b) Simulated Genotypes

To simulate various polymorphisms, we combined peak patterns of single-molecule genotypes from mononucleotide microsatellites, which themselves well approximate the genotypes of single alleles. For four experimental loci, we superimposed genotypes of two simulated alleles differing in length from 0 to 4 bases, and simulated electropherograms representing the additive product of the alleles. Unequal amplification of alleles was modeled by varying the maximum height of each allele according to likelihoods from the unequal allelic amplification prior. To model the effects of novel insertion and deletion mutations occurring during PCR amplification, individual peaks in the genotypes of simulated alleles were allowed to vary in height from single-molecule genotypes by 2×(± 0.0143 (δ = 0.00679)) of the maximal peak's height, corresponding to the distribution of signal intensities expected from mutated PCR products [9]. To represent variability introduced by the electrophoresis process, peaks were then modified by 2×(± 0.0052 (δ = 0.00744)) of the maximal peak's height [9]. 100 simulations were produced for each of the four experimental loci, and the fraction of correct calls was calculated by the Maximum Likelihood Estimate, using the exact method for calculation of 95% confidence intervals.

#### (c) Experimental Data

Genomic DNA from ten passaged subclones of the NIH 3T3 (ATCC) cell line, previously reported [9], was genotyped [see Additional File 2: **MethodsSupplement.pdf**] at four tracts (Loci 188, 321, 502, and 1292) [see Additional File 3: **Table 1.xls**]. Six independent genotypes were produced for each locus/subclone pair. Proper genotype interpretation was established by manual analysis of genotypes based on the D-value metric, an arithmetic method of determining mononucleotide repeat heterozygosity or homozygosity based [9]. Subsets of one to six of the replicates were randomly sampled and used as the basis for genotype interpretation by PeakSeeker. Summary statistics were calculated as before.

## Results and discussion

**Figure 2:**
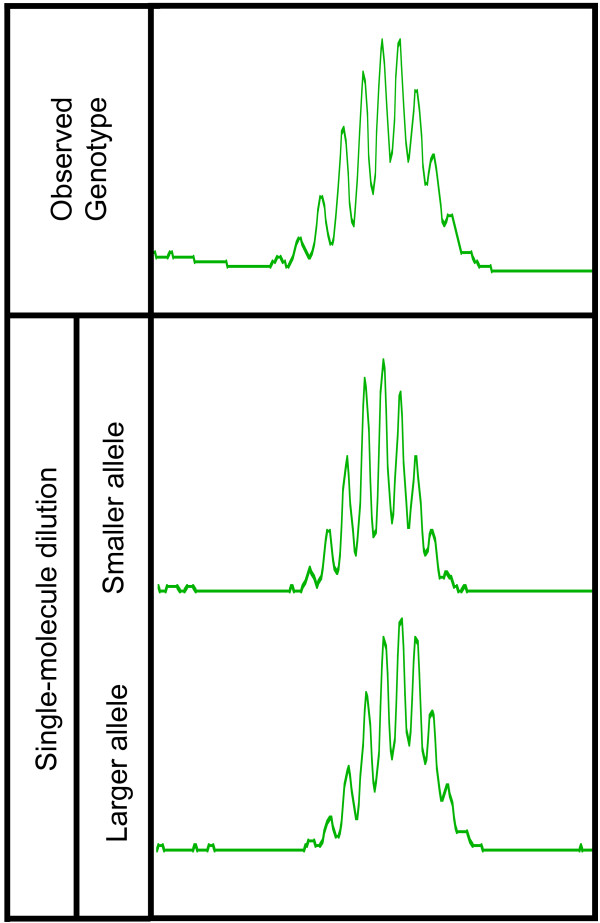
**Genotypes of microsatellite loci are the additive product of allelic genotypes**. Individual alleles of a homozygous locus can be isolated by single-molecule genotyping (bottom), which demonstrates that a genotype produced using standard quantities of genomic DNA template (top) results from the combined signals of the component alleles. Locus 321 is depicted.

Genotypes of microsatellite loci are the additive product of fluorescent signals from their component alleles: this can be demonstrated by genotyping single DNA molecules from heterozygous samples, which allows amplification each allele (Figure 2). Therefore, PeakSeeker functions by attempting to interpret electropherograms as the additive product of two homozygous genotypes, corresponding to the two alleles. Although mononucleotide markers we have examined display stutter in a roughly normal distribution, each exhibits a particular number and skew (symmetrical or asymmetrical) of peaks that appears to be marker-specific and independent of the length of the repeat tract, making it necessary to define the stutter pattern produced by a homozygote at each locus. This information can be inferred by PCR amplification of single DNA molecules isolated by limiting dilution, which approximates the genotypes of homozygotes obtained using excess template DNA [see Additional File 4: **Figure 1.pdf**]. Genotypes may differ in the relative intensity of their stutter peaks due to PCR variability [9], so we defined genotypes of homozygous loci using at least three independent single-molecule genotypes, however, peak-to-peak variability between replicates was minimal (average deviation from mean normalized peak height = 0.049, δ = 0.029). The homozygous genotype for any locus must be defined only once, so we have stored that information in a "Master File", where data for entire panels of microsatellite data can be collected for future reference.

**Figure 3:**
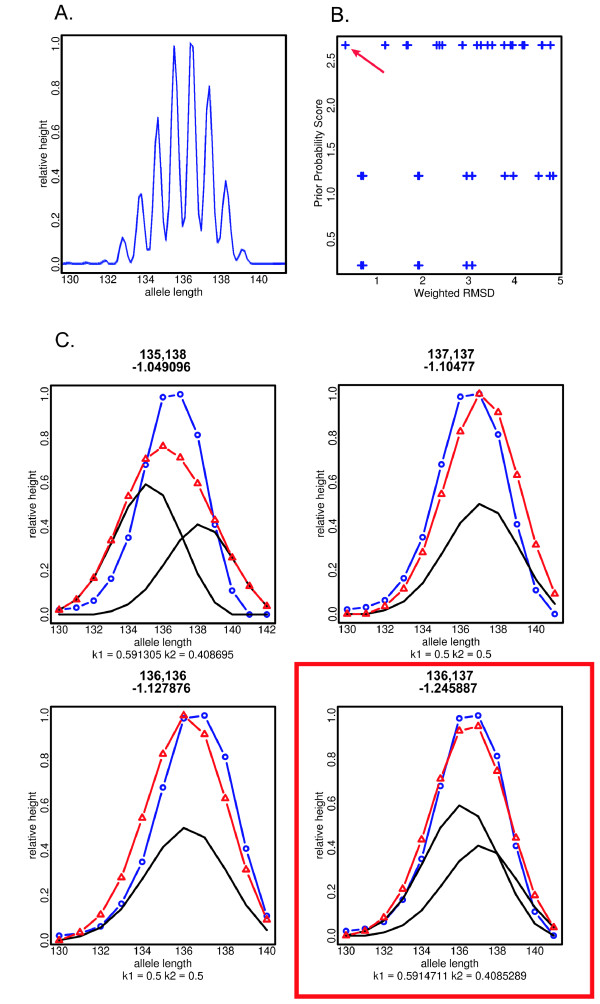
**Typical data analysis and output of PeakSeeker program**. **A**. Experimental electropherogram for locus 502. Signal intensity is displayed on the Y-axis (in arbitrary units), signal size (bp) is on the X-axis. **B**. Plot of weighted RMSD score (X-axis) versus score for likelihood of unequal allelic amplification (Y-axis) for all electropherogram interpretations considered in this example. Red arrow indicates a single interpretation that is maximal along both axes. **C**. In ascending order, panels represent the four most likely interpretations of the experimental genotype as the homozygous or heterozygous combination of two alleles. The experimental genotype is displayed in blue, with electropherogram peaks represented at the intersection of line segments (open circles). Inferred genotypes of the individual alleles are represented by black traces, and the peaks of their additive product are displayed in red (open triangles). The relative amplification of the two alleles (*K1 *and *K2*) is displayed at the bottom of each panel, with the lengths of the proposed alleles reported at the top of each panel, below which is the adjusted RMSD score for the proposed genotype interpretation. The correct call, with the lowest score, is indicated in red, and corresponds to the score indicated in panel **B**.

PeakSeeker enumerates all possible combinations of alleles that could have generated an electropherogram, then for each potential explanation, varies relative amplification of alleles to maximize the fit between experimental data and the calculated product of the individual allelic genotypes. Initial drafts of the program evaluated proposed genotype explanations by the root mean square deviation (RMSD), which measures the degree of similarity between the heights of corresponding peaks in observed data and the calculated explanation. Using that metric, the algorithm frequently proposed interpretations which fit the data well, but which required differences in relative amplification of alleles which were not empirically realistic. For example, a genotype representing a homozygote could instead be interpreted as heterozygote where one allele contributed 99.9999% of the signal, and the remaining 0.0001% from the second allele was assigned to account for some small degree of variability elsewhere in the genotype; although this interpretation fits the observed peak pattern best, given our observations about PCR amplification of diploid alleles, it is an unlikely interpretation. Thus, we have incorporated into our model a prior estimate of the relative amplification of two alleles following PCR. Based on this empirical distribution, a penalty is applied to RMSD scores that is inversely proportional to how biologically realistic the required degree of unequal allelic amplification. Combining those two metrics provides a simple, yet powerful, statistical model of PCR dynamics, and results in significantly greater differences between the scores of genotype interpretations that are biologically feasible and the scores of those that are not realistic (Figure 3B). Expectedly, the fraction of correct microsatellite interpretations is also increased under this model.

**Figure 4:**
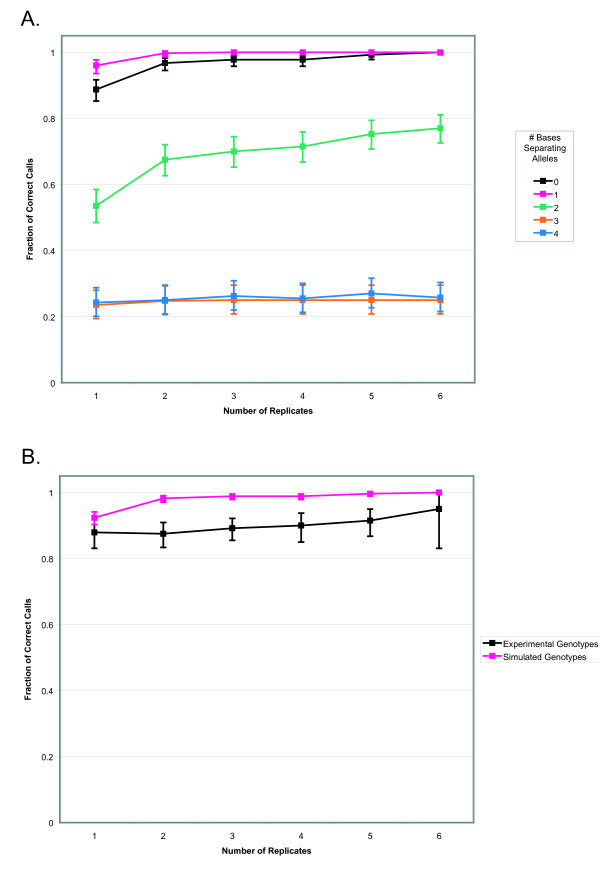
**Relationship between number of replicate genotypes performed and accuracy of data interpretation**. **A**. simulated genotypes and **B**. experimental genotype data. The fraction of correct calls for each number of replicates is displayed, error bars indicate 95% confidence interval. Simulated genotypes in **B **contain equal numbers of homozygotes and heterozygotes with alleles separated by one base.

To test the software, we designed a genotype simulation program so that the true lengths of the alleles could be controlled. Our simulations suggested that the accuracy (the fraction of correct calls compared to the known lengths of the simulated alleles) of the PeakSeeker program was proportional to the number of replicate genotypes provided for each locus (Figure 4A), with notable increases in accuracy not occurring after 3 replicates. This finding was expected, as the effects of stochastic variation from any single genotype become less pronounced as more independent replicates are incorporated into the analysis. The algorithm was most accurate in calling genotypes for locus 1292, a marker with an asymmetric distribution of stutter peaks. We find that PeakSeeker tends to work best for such markers, since the additive product of asymmetrical stutter patterns tends to give heterozygous genotypes that look vastly different from homozytgotes, whereas symmetrical alleles often yield heterozygous peak patterns that are very similar to, but simply wider than, homozygotes. PeakSeeker was also sensitive to the relative lengths of the alleles in the simulated genotypes. The highest accuracy was obtained for homozygotes and for heterozygotes with alleles separated by 1 base in length, and lesser accuracy was achieved for alleles separated by 2 bases in length. Uniformly low fractions of correct calls were observed for greater amounts of allelic separation. Nevertheless, we do not consider this to be a limiting factor, as alleles separated by three or more bases in length produce a bimodal distribution of peaks that can be readily identified by manual inspection [17](see Figure 1). Future versions of the program may incorporate a dynamic scoring method, allowing different scoring parameters to be employed when particular combinations of alleles are suspected; however, such improvements are beyond the scope of the present work.

As a functional test, we genotyped ten passaged subclones from a diploid mouse cell line [9]. To establish the proper genotype interpretation for each sample, we interrogated genotypes manually using an arithmetic method [9] unrelated to the PeakSeeker approach. Manual analysis revealed that four of the five microsatellites were polymorphic for multiple isolates, and that homozygous alleles and heterozygous alleles separated by differences of one base were represented. We then evaluated how frequently PeakSeeker correctly interpreted the electropherograms (Figure 4B). Again, the accuracy of PeakSeeker was proportional to the number of replicated genotypes used as the basis for data interpretation, although with lower accuracies than those obtained with simulated data, due to the presence of three sample/loci pairs which showed high rates of PCR error and were frequently called incorrectly. As before, locus 1292 yielded the highest accuracy, with 98.3% correct calls with only one replicate provided.

There were two instances where the results of PeakSeeker analysis and manual data analysis did not agree, but in both cases, PeakSeeker interpretation demonstrated that electropherograms were significantly different than expected from manual calls, and was therefore accepted as correct.

For the typical panel of mononucleotide microsatellites we examined here, PeakSeeker has proven well-suited to interpreting genotypes when overlap of alleles is the most significant, which are also the most difficult cases to call by eye. Thus, the program serves as a valuable augmentation to manual analysis, and can substantially increase throughput. However, if markers are selected for either limited stutter or asymmetric stutter peaks, the program should autonomously achieve perfect accuracy when a limited number of replicates are performed. The PeakSeeker algorithm could potentially be adapted to improve, and perhaps to standardize, the analysis of conventional microsatellites.

## Availability and requirements

**Project name**: PeakSeeker v1.0

**Project home page**: None, program attached as supplementary information.

**Operating system**: Platform independent (tested on Linux and OS X)

**Programming language**: Perl, R Package for Statistics

**Other requirements**: GeneMapper v4.0 software (ABI) or equivalent, the R Project for Statistical Computing

**License**: Source code and executables are freely available for academic users.

**Any restrictions to use by non-academics**: License required

## Competing interests

The authors declare that they have no competing interests.

## Authors' contributions

JT designed and implemented the software algorithm and data simulation program. SJS conceived of the study, designed the software algorithm, directed the work, performed laboratory studies, and wrote the article. Both authors read and approved the final manuscript.

## Supplementary Material

Additional File 1 **PeakSeeker_V1.** PeakSeeker v1.0 program Perl scripts and Perl libraries, with example data. Click here for file

Additional File 2 **Methods Supplement.** Description of genotyping protocol, software architecture, algorithm workflow and scoring mechanism. Click here for file

Additional File 3 **Sequence of microsatellite markers.** Oligonucleotides are listed, 5' to 3'. Click here for file

Additional File 4 **Single-molecule genotypes approximate those obtained from homozygotic samples.** Genotypes of three representative mononucleotide tracts from known homozygous samples produced using standard quantities of genomic DNA as template (red traces, from [9]), each superimposed with three genotypes derived from single DNA molecules (green or blue traces, representing labeling with HEX or 6-FAM, respectively). Click here for file

## References

[B1] Cohen H, Danin-Poleg Y, Cohen CJ, Sprecher E, Darvasi A, Kashi Y (2004). Mono-nucleotide repeats (MNRs): a neglected polymorphism for generating high density genetic maps in silico. Hum Genet.

[B2] Mukherjee M, Minal V, Mittal RD, Mittal B (2002). Allelic variation of BAT-26 and BAT-40 poly-adenine repeat loci in North Indians. Int J Mol Med.

[B3] Hughes CR, Queller DC (1993). Detection of highly polymorphic microsatellite loci in a species with little allozyme polymorphism. Mol Ecol.

[B4] Streisinger G, Okada Y, Emrich J, Newton J, Tsugita A, Terzaghi E, Inouye M (1966). Frameshift mutations and the genetic code. This paper is dedicated to Professor Theodosius Dobzhansky on the occasion of his 66th birthday. Cold Spring Harb Symp Quant Biol.

[B5] Wells RD, Dere R, Hebert ML, Napierala M, Son LS (2005). Advances in mechanisms of genetic instability related to hereditary neurological diseases. Nucleic Acids Res.

[B6] Zhang L, Yu J, Willson JK, Markowitz SD, Kinzler KW, Vogelstein B (2001). Short mononucleotide repeat sequence variability in mismatch repair-deficient cancers. Cancer Res.

[B7] Lee JS, Hanford MG, Genova JL, Farber RA (1999). Relative stabilities of dinucleotide and tetranucleotide repeats in cultured mammalian cells. Hum Mol Genet.

[B8] Boyer JC, Yamada NA, Roques CN, Hatch SB, Riess K, Farber RA (2002). Sequence dependent instability of mononucleotide microsatellites in cultured mismatch repair proficient and deficient mammalian cells. Hum Mol Genet.

[B9] Salipante SJ, Horwitz MS (2006). Phylogenetic fate mapping. Proc Natl Acad Sci USA.

[B10] Salipante SJ, Thompson JM, Horwitz MS (2008). Phylogenetic fate mapping: theoretical and experimental studies applied to the development of mouse fibroblasts. Genetics.

[B11] Clarke LA, Rebelo CS, Goncalves J, Boavida MG, Jordan P (2001). PCR amplification introduces errors into mononucleotide and dinucleotide repeat sequences. Mol Pathol.

[B12] Palsson B, Palsson F, Perlin M, Gudbjartsson H, Stefansson K, Gulcher J (1999). Using quality measures to facilitate allele calling in high-throughput genotyping. Genome Res.

[B13] Kuligina ES, Grigoriev MY, Suspitsin EN, Buslov KG, Zaitseva OA, Yatsuk OS, Lazareva YR, Togo AV, Imyanitov EN (2007). Microsatellite instability analysis of bilateral breast tumors suggests treatment-related origin of some contralateral malignancies. J Cancer Res Clin Oncol.

[B14] Sammalkorpi H, Alhopuro P, Lehtonen R, Tuimala J, Mecklin JP, Jarvinen HJ, Jiricny J, Karhu A, Aaltonen LA (2007). Background mutation frequency in microsatellite-unstable colorectal cancer. Cancer Res.

[B15] Bacher JW, Abdel Megid WM, Kent-First MG, Halberg RB (2005). Use of mononucleotide repeat markers for detection of microsatellite instability in mouse tumors. Mol Carcinog.

[B16] Hoang JM, Cottu PH, Thuille B, Salmon RJ, Thomas G, Hamelin R (1997). BAT-26, an indicator of the replication error phenotype in colorectal cancers and cell lines. Cancer Res.

[B17] Bacher JW, Flanagan LA, Smalley RL, Nassif NA, Burgart LJ, Halberg RB, Megid WM, Thibodeau SN (2004). Development of a fluorescent multiplex assay for detection of MSI-High tumors. Dis Markers.

[B18] Murphy KM, Zhang S, Geiger T, Hafez MJ, Bacher J, Berg KD, Eshleman JR (2006). Comparison of the microsatellite instability analysis system and the Bethesda panel for the determination of microsatellite instability in colorectal cancers. J Mol Diagn.

[B19] Vilkki S, Launonen V, Karhu A, Sistonen P, Vastrik I, Aaltonen LA (2002). Screening for microsatellite instability target genes in colorectal cancers. J Med Genet.

[B20] Wong YF, Cheung TH, Lo KW, Yim SF, Chan LK, Buhard O, Duval A, Chung TK, Hamelin R (2006). Detection of microsatellite instability in endometrial cancer: advantages of a panel of five mononucleotide repeats over the National Cancer Institute panel of markers. Carcinogenesis.

[B21] Huse SM, Huber JA, Morrison HG, Sogin ML, Welch DM (2007). Accuracy and quality of massively parallel DNA pyrosequencing. Genome Biol.

[B22] Bonk T, Humeny A, Gebert J, Sutter C, von Knebel Doeberitz M, Becker CM (2003). Matrix-assisted laser desorption/ionization time-of-flight mass spectrometry-based detection of microsatellite instabilities in coding DNA sequences: a novel approach to identify DNA-mismatch repair-deficient cancer cells. Clin Chem.

[B23] Sun X, Liu Y, Lutterbaugh J, Chen WD, Markowitz SD, Guo B (2006). Detection of mononucleotide repeat sequence alterations in a large background of normal DNA for screening high-frequency microsatellite instability cancers. Clin Cancer Res.

