# Supplementary material for: PeakSeeker: a program for interpreting genotypes of mononucleotide repeats
Source: BMC Res Notes. 2009 Feb 3;2:17. doi: 10.1186/1756-0500-2-17 (PMC2645428; doi:10.1186/1756-0500-2-17)
Supplement: Additional File 1 — PeakSeeker_V1. PeakSeeker v1.0 program Perl scripts and Perl libraries, with example data. [file 1756-0500-2-17-S1.zip › License_Agreement.pdf]

PeakSeeker Software
Academic License Agreement

The University of Washington ("UW") gives permission for you and your laboratory ("Institution") to use the PeakSeeker software ("Software"). Software is developed by Stephen Salipante, James Thompson, and Marshall Horwitz at UW ("Developers"). Software is a program to identify the lengths of the alleles of mononucleotide repeat microsatellites and comprises Perl scripts operating with the R statistical environment.

UW allows researchers at your Institution to run, copy, display and modify Software, for internal, non-profit research purposes, on the following conditions:

1. The Software remains at your Institution and is not published, distributed, or otherwise transferred or made available to other than Institution employees and students involved in research under your supervision.

If you wish to obtain Software for any commercial purposes, you will need to execute a separate licensing agreement with the University of Washington and pay a fee. This includes, but is not limited to, using Software to provide services to outside parties for a fee. In that case please contact:

UW TechTransfer Digital Ventures
University of Washington
(206) 616-3451
Email: license@u.washington.edu

2. You retain in Software and any modifications to Software, the copyright, trademark, or other notices pertaining to Software as provided by UW.

3. You provide UW and Developers with feedback on the use of the Software in your research, and UW and Developers are permitted to use any information you provide in making changes to the Software. All bug reports and technical questions shall be sent to: Stephen Salipante, email: stevesal@u.washington.edu.

4. You acknowledge that UW, Developers, and its licensees may develop modifications to Software that may be substantially similar to your modifications of Software, and that the UW, Developers, and its licensees shall not be constrained in any way by you in UW's or its licensees' use or management of such modifications. You acknowledge the right of the UW and Developers to prepare and publish modifications to Software that may be substantially similar or functionally equivalent to your modifications and improvements, and if you obtain patent protection for any modification or improvement to Software you agree not to allege or enjoin infringement of your patent by the UW, Developers, or by any of UW's licensees obtaining modifications or improvements to Software from the UW or the Developers.

5. If utilization of the Software results in outcomes which will be

published, please specify the version of Software you used and cite source below.

Citation here

6. Any risk associated with using the Software at your institution is with you and your Institution. Software is experimental in nature and is made available as a research courtesy "AS IS," without obligation by UW or Developers to provide accompanying services or support.

UW AND THE DEVELOPERS EXPRESSLY DISCLAIM ANY AND ALL WARRANTIES REGARDING THE SOFTWARE, WHETHER EXPRESS OR IMPLIED, INCLUDING BUT NOT LIMITED TO WARRANTIES PERTAINING TO MERCHANTABILITY OR FITNESS FOR A PARTICULAR PURPOSE.
