# Supplementary material for: PeakSeeker: a program for interpreting genotypes of mononucleotide repeats
Source: BMC Res Notes. 2009 Feb 3;2:17. doi: 10.1186/1756-0500-2-17 (PMC2645428; doi:10.1186/1756-0500-2-17)
Supplement: Additional File 2 — Methods Supplement. Description of genotyping protocol, software architecture, algorithm workflow and scoring mechanism. [file 1756-0500-2-17-S2.pdf]

Software Architecture and Workflow. PeakSeeker requires several user-provided data files in addition to processed genotype electropherograms. A series of "Library Files" specify what loci are present in multiplexed electropherograms, and what the respective dye label colors and expected amplicon size ranges (in bp) are for those loci. A "Run File" indicates which Library Files should be applied to electropherogram data files from specific genotyping runs. Lastly, a "Master File" encodes the single-allele peak pattern for all loci.

After referring to the Run File and opening an appropriate pairing of Library File and genotype data file, the program examines the indicated genotype size range for peaks of the relevant dye channel specified for each locus. Peaks in this range are initially filtered to exclude non-specific amplification products, which typically appear as single, isolated peaks that are devoid of stutter. PeakSeeker next identifies the peak in the genotype which has a size (bp) closest to an integer value, and assigns all peaks occupying contiguous positions (occurring in roughly 1 bp intervals) relative to this reference peak. To account for different amplification efficiencies in separate PCR reactions the height of each peak in the genotype is normalized to the height of the tallest peak, so that each peak height is a decimal value from zero to one.

If multiple genotyping reactions are provided for a sample/marker pairing, the program identifies such replicates on the basis of having identical sample names followed by the suffix "-rep*N*", where *N* represents an arbitrary identification number for the replicate. It then produces a "composite" genotype by averaging together the normalized intensities of peaks corresponding in position, and this composite is used for all further analyses.

PeakSeeker next refers to the Master File to obtain the appropriate single-allele genotype for each locus. Using that peak pattern, the program attempts to interpret the experimental genotype of an autosomal mononucleotide repeat as the additive product of the allelic genotypes. The program considers every possible homozygous and heterozygous combination of two alleles' genotypes over the specified size range, and for each proposed genotype a simulated peak pattern is produced based upon the single-molecule dilution data and the prior distribution of unequal allele amplification.

In addition to the table of interpreted genotypes, PeakSeeker produces several other outputs, intended to help the user assess the quality of the interpretations and to identify faulty calls. For each sample/locus pairing, a file is created graphically depicting each of the interpretations the program has considered and the corresponding adjusted RMSD score. The scoring information, only, is also printed to a text file. Lastly, a comprehensive score report is provided, containing the most likely interpretation and adjusted RMSD score for all sample/locus pairings specified by the Runfile.

Scoring mechanism:

(a) Distribution of Unequal Allelic Amplification. Stochastic processes during PCR amplification sometimes results in the unequal amplification of alleles [1]. We estimated the frequency and distribution of unequal allelic amplification by examining a polyguanine mononucleotide tract where the alleles varied enough in size so that genotypes of each allele shared no overlap (Locus 335). 23 samples of genomic DNA were obtained from passaged subclones of the mouse-derived NIH 3T3 (ATCC) cell line [1], genotyped as described, and the amplification ratio for component alleles was calculated by:

$$amplification\_ratio = max(H1, H2) / min(H1, H2)$$

where H1 and H2 are defined as the heights of the tallest peaks in the genotypes of the two individual alleles. A probability density function based on the gamma distribution was fit to a histogram of these data using least-squares fitting to estimate the scale and shape parameters of the gamma distribution, calculated as 29.801447 and 43.743393, respectively. This probability density function was used as an estimate of the probability distribution of unequal allele amplification throughout our experiments.

(b) Scoring Proposed Genotypes. The PeakSeeker program operates by attempting to interpret the genotype of an autosomal mononucleotide repeat as the additive product of two allelic genotypes. Initially, the PeakSeeker algorithm produces genotypes for proposed interpretations of experimental data by:

$$peak\_pattern(A1, A2, locus) = k1 \times pp1(A1, locus) + k2 \times pp2(A2, locus)$$

where *peak_pattern(A1,A2)* represents the additive genotype produced by combining the two component alleles, *pp1(A1)* and *pp2(A2)* are the genotypes of the individual alleles for the specific locus, where *A1* and *A2* represent the lengths of the two respective alleles, and *k1* and *k2* are constants for the relative amplification ratios of *pp1* and *pp2*. Because PCR does not always amplify alleles in equal proportions, and because the actual relative contribution of signal from two overlapping alleles cannot be known from electropherogram data, *k1* and *k2* are allowed to vary as positive numbers between zero and one.

The proposed interpretations are evaluated by measuring the Root Mean Square Deviation (RMSD) between the heights of corresponding peaks in the experimentally observed data and the proposed genotype interpretations. The RMSD metric calculates

the average deviation between the proposed and observed peak patterns, and inversely correlates with the degree of similarity between the proposed peak pattern and the experimentally observed electropherogram data:

$$RMSD = \sqrt{\sum (|proposed\_peak(i) - observed\_peak(i)|^2 \times W_i)}$$

where *proposed_peak(i)* and *observed_peak(i)* are the relative heights of individual proposed and observed peaks, respectively, and W_i represents an arbitrary "weight" factor applied to modify the relative score contribution for peaks of different heights:

$$W_i = (1.075^{observed\_peak(i)}) \times 0.5$$

The scoring method also accounts for how well a given genotype interpretation conforms to prior knowledge about PCR dynamics, as we have observed that a relatively narrow range of unequal allelic amplification is empirically realistic. Therefore, the scoring algorithm combines the RMSD with the prior probability of the proposed allelic amplification ratio:

$$f(A1, k1, A2, k2) = RMSD - prior\_score$$

This function generally increases as the fit between proposed genotype interpretations and observed data improves. Given our model of PCR dynamics, this function can be considered a density function for the frequency of the parameter set (*A1*,*k1*,*A2*,*k2*) yielding experimentally observed data.

The relative amplification of alleles for any homozygote cannot be inferred, thus, the ratio of unequal amplification for homozygotes is scored as 0.5423:0.4577, a ratio maximized from both experimental and simulated data.

All possible allelic combinations are enumerated and scored for two alleles of any size (*A1*,*A2*), and for any relative amplification ratio (*k1*,*k2*), and the interpretation with

the lowest score is accepted as correct. Because our model requires that both alleles contribute signal to the experimentally observed genotype, we assign alleles only to positions where there is a detectable electropherogram peak. In order to choose the best relative amplification ratio for a given combination of alleles, that ratio is varied in order to maximize the modified scoring function. It is possible to determine the maximum of the modified scoring density function by allowing $k1$ and $k2$ to vary between zero and one, given the constraint that $k2 = (1 - k1)$.

Genotyping. Oligonucleotides (Operon) are listed (**Supporting Information, Table 1**). All reverse primers carry the "pigtail" sequence 5'-GTTTCTT-3' to correct artifacts related to *Taq*-mediated adenylation of PCR products [2, 3]. 5 µl PCR amplifications containing roughly 9 ng genomic DNA were carried out for 40 cycles using Taq DNA polymerase (Qiagen). PCR fragments were resolved with an ABI PRISM 3730xl Genetic Analyzer equipped with POP-7 polymer and a 36 cm capillary array. Electropherograms were initially processed with GeneMapper v4.0 software (Applied Biosystems) using the AFLP analysis method with the following analysis parameters: [A] for AFLP analysis, analysis of green, blue, and yellow dyes with no normalization, analysis range from 50 to 400, alleles named using labels, and panel generated with bin width of 1.0 bp for all samples, [B] for peak detection, full range analysis and size call ranges, no smoothing, baseline window of 600 pts, peak amplitude thresholds of 50 for all channels, minimum peak half width of 2 pts, polynomial degree set to 3, peak window of size of 7 pts, with local southern size calling method, and [C] for peak morphology, a maximum peak width of 1.98 bp. Electropherogram data was written to a genotype table containing columns for the sample name and dye color,

followed by allele size and allele height (listed contiguously), with the number of alleles equaling the number of bins. The table was exported as a tab-delimitated text file.

For single-molecule genotyping, a limiting dilution of template DNA was prepared and genotyped as before, except PCR was performed for 60 cycles. The height of each peak in a genotype was normalized to that of the tallest peak, and the relative intensities of analogous peaks from three to five independent genotypes were averaged together in order to estimate the genotype of a homozygous locus. To avoid accepting genotypes with artifactual peak distributions resulting from PCR error occurring during early rounds of amplification, samples exhibiting little difference (less than 5%) between the intensity of the tallest and second tallest peaks were discarded.

References

1. Salipante SJ, Horwitz MS: **Phylogenetic fate mapping**. *Proc Natl Acad Sci U S A* 2006, **103**(14):5448-5453.
2. Brownstein MJ, Carpten JD, Smith JR: **Modulation of non-templated nucleotide addition by Taq DNA polymerase: primer modifications that facilitate genotyping**. *Biotechniques* 1996, **20**(6):1004-1006, 1008-1010.
3. Palsson B, Palsson F, Perlin M, Gudbjartsson H, Stefansson K, Gulcher J: **Using quality measures to facilitate allele calling in high-throughput genotyping**. *Genome Res* 1999, **9**(10):1002-1012.
