# Supplementary figures and images for: PeakSeeker: a program for interpreting genotypes of mononucleotide repeats
Source: BMC Res Notes. 2009 Feb 3;2:17. doi: 10.1186/1756-0500-2-17 (PMC2645428; doi:10.1186/1756-0500-2-17)

| Marker                                                                           |                                                                                    |                                                                                     |
|----------------------------------------------------------------------------------|------------------------------------------------------------------------------------|-------------------------------------------------------------------------------------|
| 188                                                                              | 321                                                                                | 502                                                                                 |
| 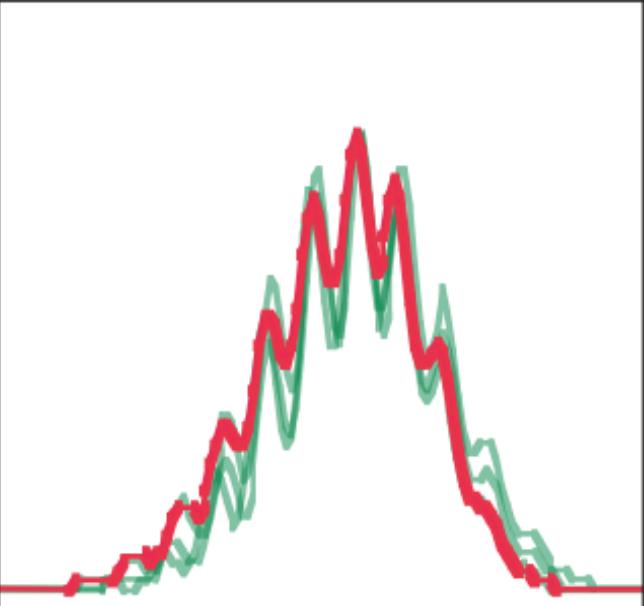 | 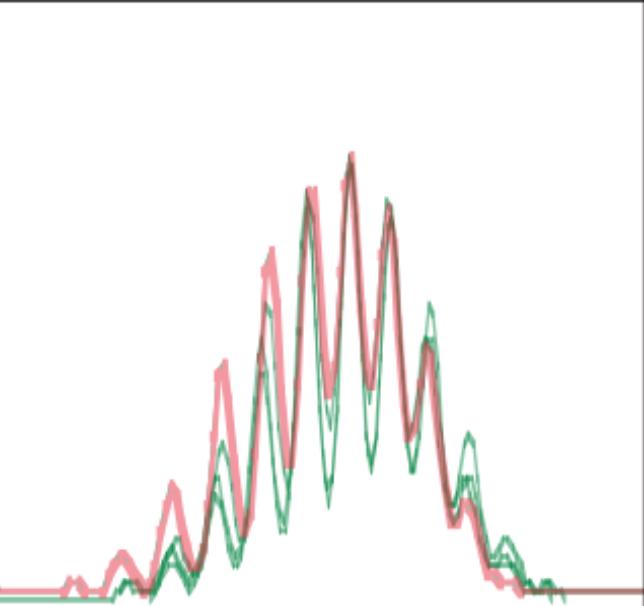 | 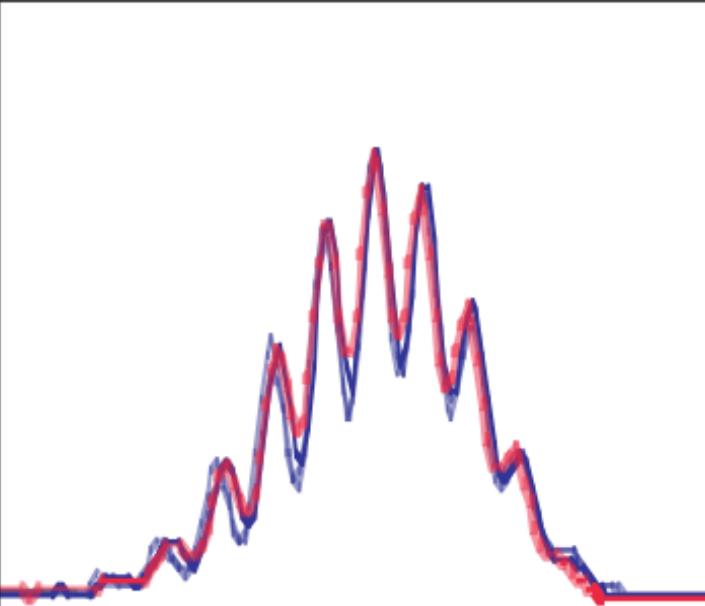 |

Supplement: Additional File 4 — Single-molecule genotypes approximate those obtained from homozygotic samples. Genotypes of three representative mononucleotide tracts from known homozygous samples produced using standard quantities of genomic DNA as template (red traces, from [9]), each superimposed with three genotypes derived from single DNA molecules (green or blue traces, representing labeling with HEX or 6-FAM, respectively). [file 1756-0500-2-17-S4.pdf]
